# A Predictive Approach for Disassembly Line Balancing Problems

**DOI:** 10.3390/s22103920

**Published:** 2022-05-22

**Authors:** Iwona Paprocka, Bożena Skołud

**Affiliations:** Department of Engineering Processes Automation and Integrated Manufacturing Systems, Faculty of Mechanical Engineering, Silesian University of Technology, Konarskiego 18A Str., 44-100 Gliwice, Poland; bozena.skolud@polsl.pl

**Keywords:** reverse logistics, product recovery, reusing, remanufacturing, disassembly line balancing, disassembly time estimation

## Abstract

In selective serial disassembly sequence planning, when the target node (component) is reached, the selective disassembly task is completed and the refurbished component is repaired, reused or remanufactured. Since the efficient utilization of existing resources is necessary, it is crucial to predict disassembly operation times and the condition of joints for recycling, reusing or remanufacturing. The method of estimating the disassembly times of a joint if it is intended for remanufacturing, recycling and reuse is an important and urgent requirement for research development and results. The aim of the paper is to investigate the disassembly system with predicted operation times and the quality of product connections (joints) in order to balance the line smoothness index, to minimize a line time factor, line efficiency and profit and minimize an ex post error. Disassembly times for remanufacturing, recycling and reuse are estimated separately based on the historical data of disassembly times and the quality of joints. The presented estimation method of disassembly operation times increases the reliability and efficiency of elaborated balances of tasks in lines. Underestimated disassembly operation times can be compensated for during the idle points in the successive cycles, provided that the transport operations are performed manually and that travel time determines the cycle time.

## 1. Introduction

Today, enterprises support a circular economy strategy by adopting closed-loop life cycle models for their products [1]. Recovering materials, especially valuable materials, has become an obligation due to limited natural resources of the Earth. The proper recycling and utilization of harmful and hazardous substances contained in waste electronic and electric equipment significantly reduces environmental pollution. The recovered raw material can be passed on producers, steel mills and foundries for further processing.

Harmful and dangerous substances include mercury; lead; bromine compounds; cadmium; barium; and compounds containing halogens, such as PCB (Polychlorinated Biphenyls), asbestos and freon. The European Parliament has adopted a directive aimed at reducing the negative impact of the above-mentioned materials on the environment. The use of these substances in devices classified as electrical and electronic equipment was banned. On the other hand, companies dealing in sales and imports were obligated to collect and process used equipment. In the directive, a minimum level for material recovery and recycling was also specified [2]. The list of various regulations concerning the prevention and protection of the earth’s environment was provided in [3].

Products are recovered in order to obtain raw materials or semi-finished products for production through recycling, disassembly, sorting and reengineering. Effective use of the disassembly line is associated with solving the problems of planning (when to start an order), sequencing (determines the disassembly sequence of models), scheduling disassembly tasks (in what order to perform tasks on the stands) and balancing the disassembly line (assigning tasks to workstations). In the problem of balancing the disassembly line (DLM), the operations (tasks) between the disassembly line stations are evenly distributed so that the idle time of the machines is as short as possible. Before starting the balancing of the disassembly line, the following conditions must be met [4]: the times of performing operational tasks on machines are known, the sequence relations between operations are known and the size of the production cycle or the number of machines is known. The result of balancing is to designate the fewest workstations at a given cycle time or determining the lowest cycle value with a given number of stations.

Although the disassembly operations are expensive, the lines are often not dedicated to one type of product. Fixed disassembly lines increase productivity and throughput but also increase the cost of unused capacity. In the age of globalization, there is a tendency to increase the flexibility of the disassembly line in order to adapt orders belonging to a certain product family. Due to the high demand for the disassembly of various products, managers face the problem of an increasing variety of disassembly products on the lines. Cloud production enables efficient order taking and the full use of the disassembly line resources [5]. The necessity of proper line balancing and sequencing is presented in [6].

The assembly and disassembly line balancing tasks may seem similar but closer study reveals the differences between these processes. The disassembly process is not the reverse of the assembly process as the product is often damaged at the end of its life cycle, components are missing and disconnection of the used joints over time may be difficult due to wear, rust or deformation. The problems of balancing the disassembly lines are made difficult due to possible variations in operation times but also the need to use variable tools in the event of disassembly difficulties. In the assembly process, there is no doubt about the quantity, quality and consumption of the elements used. In the disassembly process, both the wear and the quality of the disassembled product should be taken into account. In the case of assembly, it is possible to predict operation times with high accuracy, without taking into account the experience of the personnel performing the operations. In the case of disassembly, component wear may extend the process or require the use of additional tools [7].

Due to the unknown quality of joints, the times of disassembly operations are stochastic, not deterministic. Therefore, in order to estimate the times of the disassembly operations, it is necessary to analyze the historical states of the joints and the times of their disassembly. The primary objective of this paper is to minimize the number of disassembly stations for a given cycle time of the disassembly process. The second objective is to estimate disassembly operation times using historical information on joint conditions and disassembly times of the models (products) received from the reverse logistics network. Estimating both connection states and disassembly times gains value rather than deriving mean times without learning from the past.

The rest of the paper is organized as follows: the literature review is presented in Section 1.1. The model of a disassembly balancing line for a single product is presented in Section 2. The estimation method of disassembly operation times is presented in Section 3. The comparison of the balances obtained through the use of heuristic methods for estimated disassembly times, together with necessary analysis, is presented in Section 4. The paper ends with a brief summary of the results and future research objectives (Section 5).

### 1.1. Literature Review

#### 1.1.1. Disassembly Planning Problems

In the literature, the problems of planning the disassembly sequence for production lines dedicated to a single product, mixed products and multiple products are identified. Three variants of the disassembly systems have been identified [8]: a single disassembly station, a disassembly cell or a disassembly line. Disassembly lines are preferred for high productivity levels and high volumes of a product.

Let us analyze the problem of disassembly lines dedicated to individual products. Kheder et al. [9] assigned the disassembly operations of a single product to stations, assuming that each component of the disassembly mechanism was a rigid solid and the connections between parts were perfect. Marconi et al. proposed the model of estimating disassembly time based on the actual conditions of a product and its components, such as deformation, rust and wear, using corrective factors [1]. Liu et al. [5] estimated the corrective factors for the disassembly tool based on the data mining process of the product geometrical features. Total disassembly time depends on the basic disassembly time, disassembly direction changes, disassembly tool changes and moving time between disassembly points. Two case studies were performed for the verification of the method: a washing machine and a coffee machine. Additionally, the disassembly sequence planning problems for a single product are considered due to the complexity of the process, including tool changes and disassembly directions.

Let us analyze the disassembly problems of mixed models, in which a product with various components and subassemblies is disassembled. The state-based approach includes multiple possible disassembly paths in an AND/OR graph [10]. Finally, only one general disassembly path is selected for the stochastic disassembly balancing problem for all variants of the product. For example, Ilgin et al. [8] presented a balance for a mixed-model disassembly line for traditional and smart phones. Riggs et al. [11] proposed a joint (single) precedence graph based on the different end of life states of a product. The disassembly job for each component of a product is represented as a node in the precedence graph. All operation times related to the removal of a component are summed up in one time when multiple tasks are required to remove the component. In the stochastic disassembly line balancing problem each task time is a normal random variable with the total time variance for each workstation below a predefined value. Additionally, Boysen et al. [12] reduced the mixed-model balancing problem to a single-model case, in which the balancing problem is solved. Models represent all variations of the same base product that differs in specific customizable product attributes. The joint precedence graph is formed based on the models of product variants using pairs of triangular XOR nodes, which express that the connected options are mutually exclusive. Operator “Xor” (Exclusive Or) performs a logical operation between its inputs. Operator returns true if either input is true, but false if both are true or neither is true. “Xor” means that only one of the disassembly options has to be selected. The expected joint disassembly times are based on probability of product versions appearance and task times.

A popular strategy for solving balancing problems with mixed-model disassembly lines is to build a single disassembly model for different component variants with an average runtime. The disadvantage is the idle machines that are not used in every variant of the product. A disassembly model represented by a common graph for mixed models and based on average task times and general disassembly stations is not sufficient when high station performance is required.

Let us analyze the disassembly problems of multi-products. An example of the multi-product disassembly line dedicated to refrigerators and washing machines is presented in [13]. Regular products are what customers and downstream disassembly stations require, with an exponential distribution of times between arrivals. The disassembly process is triggered by a single Kanban. The speed of delivery depends on the average time of disassembly at the station, probability of a missing or defective item. Kanban is a workflow management method for defining, managing and improving production and services [14]. The word “Kanban” stands for a visual signal. The Kanban methodology uses visualization to track and reduce work in progress and maximizes the workflow. Kanban implementation means using Kanban boards and cards to visualize workflows. The interrelated problem of line balancing and model sequencing is presented in [15]. Models arriving at an average rate are disassembled on the U-shaped line. The precedence relationships of each model are presented in a combined precedence graph with no model relationships to increase the number of possible alternatives. A task is assigned to a disassembly station with a certain probability. If the probability is less than the threshold set for the assignment of the task to the disassembly station the task is assigned to the corresponding station.

The related literature (approximately 96% of the studies) considers mainly the single-product case due to the complexity of the problem [16]. However, single-product disassembly line balancing problems are often optimally solved and an interest in the research of a given variety of products is noticeable. Mixed-model disassembly and multi-product disassembly problems are more practical. In fact, building a mixed model for a number of products creates a production line dedicated to the products. Although a disassembly line is dedicated to a mixed model of products, the first product is disassembled at a time until the product is completely disassembled, and then the second product is disassembled [8]. When disassembling one product, only the necessary stations are on and the remaining are idle at that time.

In the field of engineering, disassembly times are mostly predefined, assuming that each component of the disassembly mechanism is a rigid solid and the connections between parts are perfect [9]. Additionally, disassembly times are assumed to be described by normal [11,17] or exponential distributions [18], observed standard average times [13,19]. Mandolini [17] proposed an analytical method for estimating the disassembly time in a complex assembly. In order to estimate the disassembly time, the data on the main assembly liaisons and the correction factors were analyzed. For example, the corrective factor can depend on deformation, rust and wear, which encompass the actual conditions of a product [1]. Liu et al. [5] estimated the corrective factors for disassembly tools based on the data mining process of product geometrical features. Francia et al. [20] defined parameters, which allowed one to best describe the dismantling operation of an element: the use of force, disassembly mechanism, the use of tools, the repetition of the parts, the recognition of the joints, product structure, accessibility, positioning and basic time. By performing the sum of these values, an evaluation of the operation under examination was obtained, expressed in time measurement units (TMUs). This was combined with the classification of using/disposing options after disassembling them from the assembly; or how to allocate the recycling of elements such as precious metals, plastic materials and elements that cannot be used; or how to regenerate processes and reintegrate them with the production cycle; or how best to do maintenance. The time of operation was evaluated as 10 × TMU × 0.036, where 0.036 is the standard value of conversion from a TMUs task to seconds [21,22].

The main advantage and distinguishing approach of this paper is using historical information data on joint variants for recycling, reusing or remanufacturing and disassembly times in order to estimate disassembly operation time. Most existing approaches include the actual conditions of a product to select the corrective factors for estimating the disassembly time. In our approach, the estimated joint disassembly times are based on the probability of the connection variant and the estimated task execution time, taking into account historical time data for each connection variant separately. The most similar approach is presented in [12], where the estimated times of disassembly of connections are based on the probability of the product version appearance and the times of execution of tasks. The difference is in the product version, not in the joint variant. The difference is also in the probability method of a joint version and the method of estimating disassembly times.

The method of the time estimation of disassembly operations presented in this paper is universal and can be applied either to single product disassembly problems or mixed and multi-product disassembly problems after receiving a common joint graph. In the case of multiple or mixed model problems, historical data is grouped dependent on the station and joint variant (recycling, reusing or remanufacturing).

#### 1.1.2. Disassembly Planning Methodologies

When analyzing methodologies developed for assembly and disassembly line balancing problems, three groups can be classified: heuristic procedures, meta-heuristics and mathematical programming techniques. The simplest heuristic method is the largest candidate rule (LCR) [19,23]. It consists of assigning tasks to a disassembly station from the longest to the shortest [24]. Another heuristic method is the ranked positional weight (RPW) method [13]. In the Kilbride and Wester method, operations with the fewest predecessors are assigned to the workstation first [25]. This method assigns tasks to stations based on the importance of the task. The algorithm of the immediate update first-fit (IUFF) heuristic is presented in [26]. Additionally, Gungor and Gupta [27] and Tang and Zhou [28] proposed heuristic solution procedures. Marconi et al. concluded in their paper [1] that the priority rule of the minimum number of disassembly operation does not reach the best sequence.

The second group of methods includes metaheuristics, e.g., the ant colony optimization algorithm for optimizing disassembly sequence planning, proposed by Kheder et al. [9]. Agrawal and Tiwari [15] proposed the ant colony optimization algorithm to address balancing and sequencing problems. Liu et al. [5] proposed the discrete bees algorithm.

The papers in the last group developed techniques for dynamic programming and integer programming [29], fuzzy goal programming [30], linear physical programming [8] and mixed integer nonlinear programming [3]. For a comprehensive review of the literature on disassembly line balancing methodologies, see [16].

The choice of the method of balancing disassembly tasks affects the quality of the solutions obtained, but the scope of the article is limited to estimating the time of disassembly tasks and the effective allocation of tasks to disassembly stations will be the subject of future works.

### 1.2. Goals and Approaches

The highlight of this paper is listed as follows:

− The problem of balancing the disassembly of a single-product line with three possible cases of joint disassembly is investigated: recycling, reuse or regeneration (remanufacturing) and a fourth possible case of waste. The presented method of estimating disassembly times is important for the research development and results from an urgent need [16].
− A method of estimation disassembly operation times based on the analysis of historical information on the quality of a product joints and disassembly times is proposed. The objective is to minimize the number of assembly stations for the predicted times of disassembly operations and predefined cycle time. Another objective is to maximize the disassembly line efficiency, line smoothness index and line time.

## 2. Problem Definition, Assumptions and Notation

The aim of the paper is to investigate the disassembly system with predicted operation times and the quality of model connections (joints) in order to balance the line smoothness index, to minimize line time factor and ex post error and to maximize line efficiency and profit. The problem is to estimate the disassembly times of each model and maximize the profit of the disassembly line. Disassembly times are estimated from historical data on disassembly times for each product joint separately for reproduction, recycling and reuse.

The disassembly line is dedicated to the number of products *d^i^* of the same type i, i = 1,…, d^i^ with the given constrains:

1. each joint (assembly mechanism) $n_{j}^{i},\;j=1,\ldots ,a^{i}$ is not supposed to be perfect, joint attributes between elements are unknown,
2. the priority relation between joints describes a spatial bond relation (joint disassembly priority),
3. disassembly operations are assigned to resources in order to fully utilize disassembly capacities, taking into account the relationship of spatial constraints and cycle time constraints,
4. each product joint is disassembled for either remanufacturing, reusing or recycling. When a product joint does not qualify for one of the three disassembly options, it is dedicated as waste,
5. the historical disassembly times and quality of joints (topological and geometrical data on joints) for either remanufacturing, reusing or recycling are known. Disassembly times are predicted based on the analysis of historical information on quality of joints and disassembly times,
6. each worker is allocated to one disassembly station s, s = 1,…,b, in other words, the number of employees is equal to the number of disassembly stations b,
7. joints can be disassembled at any station without restrictions due to the tool equipment, employee skills and type of disassembly station,
8. conveyor transport times, distance between stations is predefined on the disassembly line,
9. inter-operational buffers are not allocated.

Each product i = 1,…, d^i^ is described by the disassembly graph model [31], $G^{i}=\left\{N^{i},E^{i}\right\}$, where set $N^{i}=\left\{n_{1}^{i},n_{2}^{i},\ldots ,n_{a}^{i}\right\}$ describes disassembly joints represented by nodes in a graph. Edge set $E^{i}=\left\{e_{j,k}^{i}\right\}$ describes the relationship constraints between successive nodes, $n_{j}^{i}$ and $n_{k}^{i},$ where $j,k=1,\ldots ,a^{i}-1$. Joint precedence graphs under high product variety are given in [12]. Additionally, the matrix of constraints ${\mathrm{MP}}^{i}={\left(p_{j,k}^{i}\right)}_{a^{i}\times a^{i}}$ represents the disassembly operation = joint priorities; in other words, the relationships between the successive nodes $n_{j}^{i}$ and $n_{k}^{i}$:


(1) $$p_{j,k}^{i}=\{\begin{array}{c} 1,-x,x,-y,y_{}\\ 0, \end{array}$$

Disassembly operation = joint $n_{j}^{i}$ takes precedence over $n_{k}^{i}$ (with the disassembly direction −x,x,−y,y).

The matrix of disassembly task times ${\mathrm{MT}}^{i}={\left(t_{j}^{i}\right)}_{\left(a^{i}\right)\times \left(d^{i}\right)}$ represents the disassembly operation times, $t_{j}^{i}\in ℜ$, which depend on historical information about the operation times and quality of disassembly joints.

(2) $$t_{j}^{i}=\{\begin{array}{c} ℜ{,}_{}\\ 0, \end{array}\;\mathrm{Duration}\;\mathrm{of}\;\mathrm{disassembly}\;\mathrm{operation}\;n_{j}^{i},$$

The matrix of a product batch size ${\mathrm{MZ}}^{i}={\left(z_{}^{i}\right)}_{\left(1\right)\times \left(d^{i}\right)}$ represents the batch size of product i required by the customer.

The cycle time is computed by dividing the available time D^i^ and the batch size Z^i^:

(3) $$C^{i}=\frac{D^{i}}{Z^{i}},{\;\mathrm{where}\;\max\;t}_{j}^{i}\;\leq D^{i}\leq \overset{a^{i}}{\underset{j=1}{\sum }}t_{j}^{i}.$$

The disassembly cycle cannot be exceeded, therefore a predicted method with a low ex post error is essential.

There are three disassembly options for each product joint: materials recycling and semi-products for reusing or remanufacturing. It is assumed that the costs of disassembly operations for recycling, reusing and remanufacturing are predefined as:

(4) $${\mathrm{dc}}_{j}^{i}=\{\begin{array}{c} {\mathrm{dc}\;\mathrm{rec}}_{j}^{i}\\ {\mathrm{dc}\;\mathrm{reu}}_{j}^{i}\\ {\mathrm{dc}\;\mathrm{rem}}_{j}^{i} \end{array}$$

the cost of disassembly operation $n_{j}^{i}$ in the case of recycling, reusing or remanufacturing. In the case of waste, the cost is also predefined as ${\mathrm{dc}\;\mathrm{wast}}_{j}^{i}$.

Revenues achieved after disassembling joint j of product i and selling for recycling, reusing and remanufacturing are predefined as:

(5) $${\mathrm{dp}}_{j}^{i}=\{\begin{array}{c} {\mathrm{dp}\;\mathrm{rec}}_{j}^{i}\\ {\mathrm{dp}\;\mathrm{reu}}_{j}^{i}\\ {\mathrm{dp}\;\mathrm{rem}}_{j}^{i} \end{array}$$

revenues after the disassembly of the operation j of product i, in the case of recycling, reusing or remanufacturing. In the case of waste, the revenue is ${\mathrm{dp}\;\mathrm{wast}}_{j}^{i}=0$.

**Figure 1:**
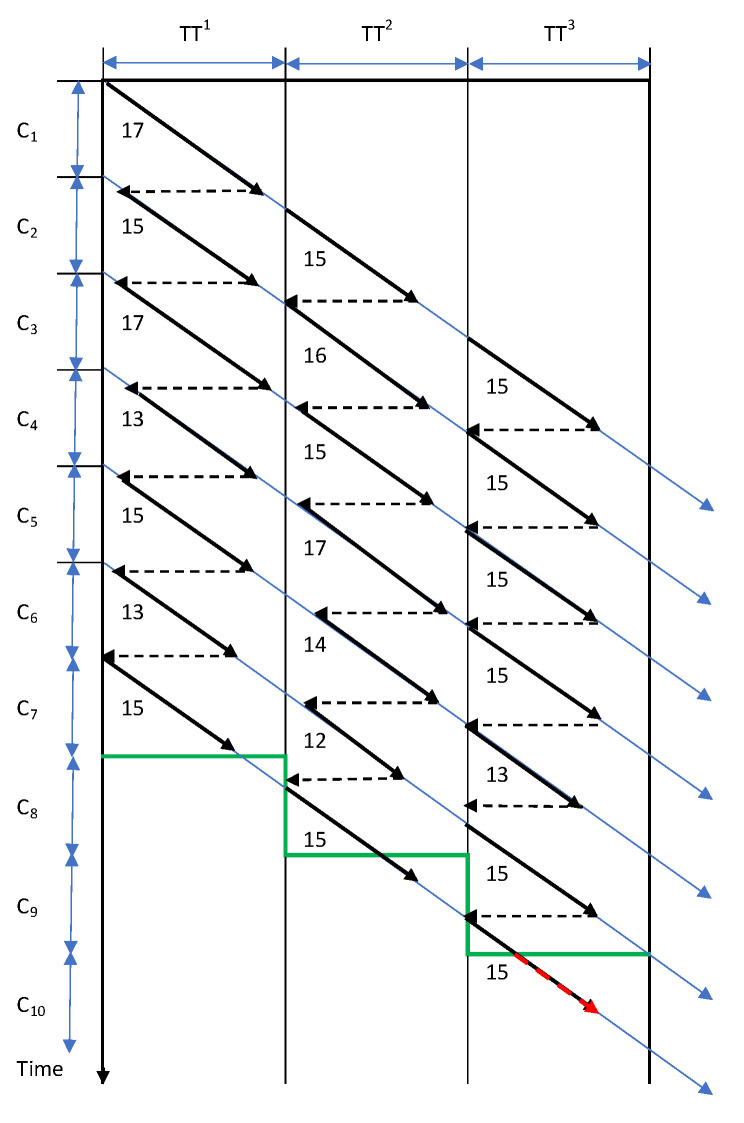
The diagram of employee/conveyor movement with the assumed disassembly cycle time C = 15, travel cycle time TT = 25 and real disassembly cycle times (12–17).

The distance and travel time of each conveyor/employee are necessary to bring into the optimization process when the travel time exceeds the cycle time [32]. Additionally, the underestimated disassembly operation times can affect the cycle time. Underestimated disassembly operation times can be compensated for by the idle times of the successive cycle times provided that the transport operations are performed manually, and travel time determines the cycle time (Figure 1). Overestimated disassembly operation times increase the cost of unused resource capacity.

Let us analyze the disassembly system consisting of three disassembly stations, s = 1,2,3, cycle time C equals to 15 min, the transport time of the conveyor between each disassembly station TT^s^ = 25 min and a batch size of the product equal to Z^1^ = 7. Finally, the disassembly time of the seven products is 10 min longer than the time achieved without taking into account underestimated or overestimated disassembly operation times (red line in Figure 1).

**Figure 2:**
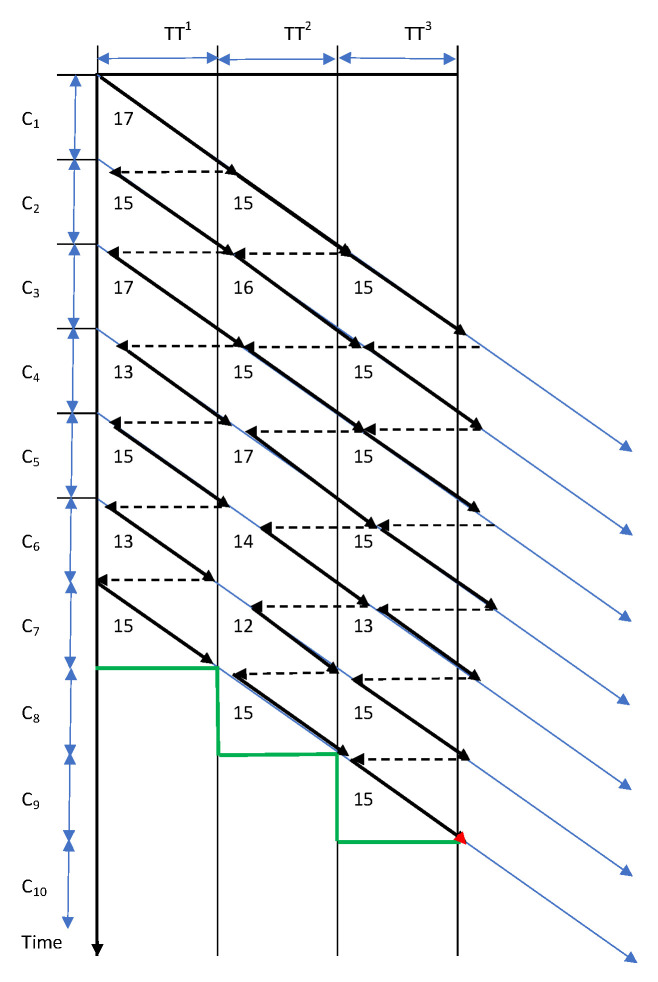
The diagram of employee movement with predicted (C = 15) and real cycle times (12, 13, 14, 15, 17), the product disassembly cycle time is equal to the transport cycle time (TT = 15).

The disassembly time of the seven products is only one minute longer for the conveyor transport time between disassembly stations, TT^s^ = 15 min and for the same differences between the assumed and real cycle times (Figure 2). Conveyor transport is performed manually, and travel time is equal to the disassembly cycle time.

**Figure 3:**
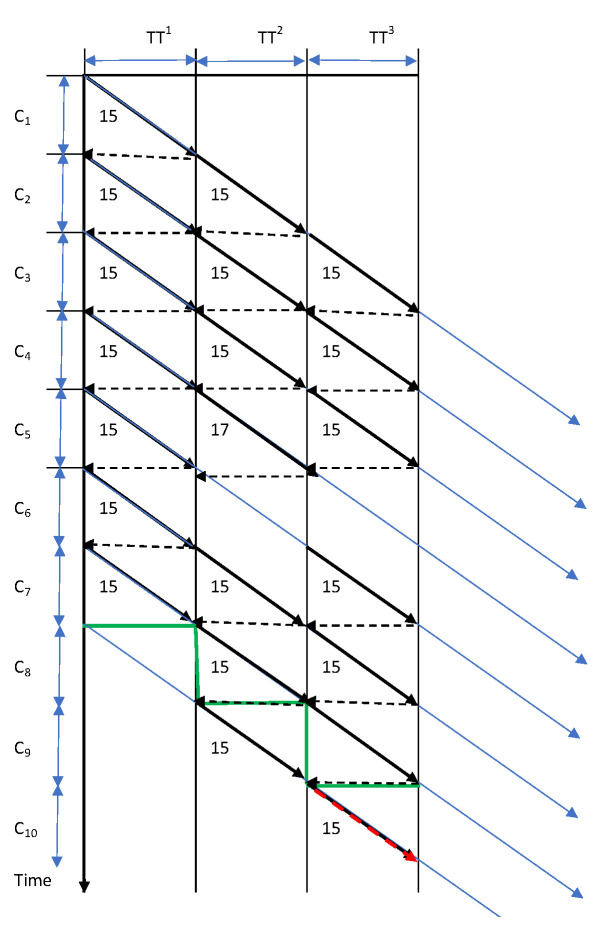
The diagram of employee movement with predicted (C = 15) and real disassembly cycle times (15, 17), transport cycle time (TT = 15) and when the product disassembly and/or transport operations are automated.

When conveyor transport and/or disassembly operations are performed automatically, any disturbance results in suspending one cycle (Figure 3). The disassembly time of a batch of seven products is increased by 15 due to a disturbance of the fourth batch of the product in the second station (red line in Figure 3).

Finally, it is assumed that the penalty cost given for late disassembly of product i due to disturbance is predefined as:

(6) $${\mathrm{rct}}^{i}=\{\begin{array}{c} {\mathrm{rc}\;\mathrm{undert}}_{}^{i}\\ 0 \end{array}$$

the cost of underestimating the cycle time of product i for each unit of time.

Otherwise, the estimation method of disassembly operation times is presented in the next Section.

## 3. Estimating Disassembly Operation Time, Cycle Time and System Efficiency

The time of the disassembly of the product joint depends on the intended use of the joint: recycling, reuse or regeneration; therefore, the data on joint disassembly times are collected separately for the three above events. The following subsections are organized to include:

- the estimation of the probability of the quality of the disassembled joint,
- the estimation of the average disassembly time of the joint,
- the estimation of the disassembly line efficiency indicators.

### 3.1. The Estimation of Disassembly Operation Time $t_{j}^{i}$

We consider a disassembly line, taking into account historical disassembly times of the product joints (operations). The duration of disassembly operation varies over time due to missing components and different joint conditions caused by wear, rust or deformation. It is assumed that the duration $t_{j}^{i}$ of each disassembly joint n^i^_j_, $j=1,\ldots ,a^{i}$ of product i, i = 1 …, d is described by truncated normal distribution with parameters changing with time. The normal distributions are popularly used to describe manufacturing processes, e.g., in the six sigma process measurement method, the goal is to achieve 3.4 defects per million opportunities (DPMO, defects per million opportunities) [33,34]. Disassembly operation time predictions are built based on historical operation times in a certain number of periods of the same length. Therefore, the disassembly system is monitored on *r* successive time periods (7),

(7) [0,D),[D,2D),…,[(r−1)D,rD),

of the same durations (a day, a shift, etc.), for which the information on the duration of disassembly operation was collected. The prediction of the disassembly system behavior was built for the next planning period [rD, (r + 1)D). We assume that the duration of the disassembly operation X^i^_j,q,1_,…,X^i^_j,q,Bq_ in the q-th historical period [(q−1)D, qD), q = 1,…, r, would have normal distributions with parameters m ∈ R and σ > 0, truncated to the positive half axis. The value B_q_ represents the number of cycles computed for the q-th period. Let us note that the CDF (=cumulative distribution function) F(⋅) of the normal distribution has the form (8),

(8) $$F\left(ł\right)=P\left\{X<ł|X>0\right\}=\frac{P\left\{0<X<ł\right\}}{P\left\{X>0\right\}}=\frac{\Phi \left(\frac{ł-m}{\sigma }\right)-\Phi \left(-\frac{m}{\sigma }\right)}{1-\Phi \left(-\frac{m}{\sigma }\right)}{,}_{}ł>0,$$

where ϕ(⋅) stands for the CDF of the standard normal distribution and ł is normally distributed with parameters m ∈ R and σ > 0. Differentiating the expression above due to the variable ł, the PDF (= probability density function) f_q_(⋅) of variable X_q,k_, is achieved, where k_q_ = 1,…, B_q_, has the following form (9):

(9) $$f_{q}\left(ł\right)=\frac{1}{\sigma_{q}\left[1-\Phi \left(-\frac{m_{q}}{\sigma_{q}}\right)\right]}\phi \left(\frac{ł-m_{q}}{\sigma_{q}}\right){,}_{}ł>0,$$

where $\phi \left(ł\right)=\frac{1}{\sqrt{2\pi }}e^{-{ł}^{2}/2}$ denotes the PDF of the standard normal distribution and m_q_, σ_q_ are parameters which change with the number of period q.

The mean value E(X_q,k_) and the variance Var(X_q,k_) of the normal distribution described by parameters m_q_ and σ_q_, with the PDF defined in (9), are given in the formulas (10) and (11):

(10) $$E\left(X_{q,k}\right)=m_{q}+\sigma_{q}\frac{\phi \left(-\frac{m_{q}}{\sigma_{q}}\right)}{1-\Phi \left(-\frac{m_{q}}{\sigma_{q}}\right)},$$

(11) $$\mathrm{Var}\left(X_{q,k}\right)=\sigma_{q}^{2}\left[1-\frac{\frac{m_{q}}{\sigma_{q}}\phi \left(-\frac{m_{q}}{\sigma_{q}}\right)}{1-\Phi \left(-\frac{m_{q}}{\sigma_{q}}\right)}-{\left(\frac{\phi \left(-\frac{m_{q}}{\sigma_{q}}\right)}{1-\Phi \left(-\frac{m_{q}}{\sigma_{q}}\right)}\right)}^{2}\right],$$

where k = 1,…, B_q_.

Each disassembly operation time X_q,k_ was monitored in each cycle k = 1,…,b_q_ for any historical period q = 1,…, r from [0,D), [D,2D),…, [(r−1)D, rD). Value b_q_ was the number of historical cycle times, and finally, the following observations were noticed:

(12) $$\left(x_{1,1},x_{1,2},\ldots ,x_{1,b_{1}}\right),\ldots ,\left(x_{r,1},x_{r,2},\ldots ,x_{r,b_{r}}\right)$$

Now, we consider first historical period [0,D), the maximum likelihood principle can be used to estimate parameters m_i_, σ_i_ of truncated normal distribution as data on x_1,1_, x_1,2_, …, x_1,b1_ are independent and identically distributed random variables.

By achieving σ_1_ given from the Equation (13) and introducing it to the Equation (14), m_1_ is eliminated numerically, using one of the approximations of the cumulative distribution functions and the PDF of the standard normal distribution.

(13) $$\sigma_{1}^{2}=\frac{m_{1}\sum_{k=1}^{n_{1}}\left(x_{1,k}-m_{1}\right)+\sum_{k=1}^{n_{1}}{\left(x_{1,k}-m_{1}\right)}^{2}}{n_{1}}=\frac{\sum_{k=1}^{n_{1}}x_{1,k}^{2}-m_{1}\sum_{k=1}^{n_{1}}x_{1,k}}{n_{1}}.$$

(14) $$-n_{1}\sigma_{1}\phi \left(-\frac{m_{1}}{\sigma_{1}}\right)+\left[1-\Phi \left(-\frac{m_{1}}{\sigma_{1}}\right)\right]\overset{n_{1}}{\underset{k=1}{\sum }}\left(x_{1,k}-m_{1}\right)=0.$$

By introducing the Maclaurin expansion,

(15) $$\phi \left(x\right)\approx \frac{1}{\sqrt{2\pi }}\left(1-\frac{x^{2}}{2}+\frac{x^{4}}{8}\right)$$

into (14), estimators ${\hat{m}}_{1}{,}_{\;}\ldots ,{\hat{m}}_{r}$ and ${\hat{\sigma }}_{1},\;\ldots ,{\hat{\sigma }}_{r}$ are achieved. By using the regression method, values ${\hat{m}}_{r+1}$ and ${\hat{\sigma }}_{r+1}$ are extrapolated for the next available period [rD, (r + 1) D) for which we have no observation.

### 3.2. The Historical-Dependent Disassembled Joint Quality of the Product

The end-of-life product is delivered with quality that changes over time. In order to estimate the quality of the end-of-life product, historical information on its mechanical and topological properties should be collected. Product quality (e.g., the conditions of joints: reversable or irreversible) influences the efficiency and profit of the disassembly line, thus, such information as joints for recycling (Rec), joints for reusing (Reu) or joints for remanufacturing (Rem) is key to planning joint disassembly duration.

Based on historical information, a quality standard (norm) is established, which classifies a given joint, as to whether it is intended for recycling or reusing (due to a high percentage of cases). The environmental conditions of the joint operation (temperature, dust, humidity), the age of the joint and the joint material affect the irreversible connection of the joint. The model of predicting the joint quality of the disassembled product can also be based on the comparative analysis of historical mechanical and topological properties of disassembled joints and their intendent use: joints for recycling (Rec), for reusing (Reu) or for remanufacturing (Rem).

Let us assume that quantities Rec, Reu and Rem are random variables with truncated normal distributions with the parameters (mean value and standard deviation) (m_Rec_, σ_Rec_), (m_Reu_, σ_Reu_) and (m_Rem_, σ_Rem_), respectively:

(16) $$\mathrm{Rec}\left(ł\right)≝P\left\{\mathrm{Rec}<ł|\mathrm{Rec}>0\right\}=\frac{P\left\{0<\mathrm{Rec}<ł\right\}}{P\left\{\mathrm{Rec}>0\right\}}=\frac{\Phi \left(\frac{ł-m_{\mathrm{Rec}}}{\sigma_{\mathrm{Rec}}}\right)-\Phi \left(-\frac{m_{\mathrm{Rec}}}{\sigma_{\mathrm{Rec}}}\right)}{1-\Phi \left(-\frac{m_{\mathrm{Rec}}}{\sigma_{\mathrm{Rec}}}\right)},$$

(17) $$\mathrm{Reu}\left(ł\right)≝P\left\{\mathrm{Reu}<ł|\mathrm{Reu}>0\right\}=\frac{P\left\{0<\mathrm{Reu}<ł\right\}}{P\left\{\mathrm{Reu}>0\right\}}=\frac{\Phi \left(\frac{ł-m_{\mathrm{Reu}}}{\sigma_{\mathrm{Reu}}}\right)-\Phi \left(-\frac{m_{\mathrm{Reu}}}{\sigma_{\mathrm{Reu}}}\right)}{1-\Phi \left(-\frac{m_{\mathrm{Reu}}}{\sigma_{\mathrm{Reu}}}\right)},$$

(18) $$\mathrm{Rem}\left(ł\right)≝P\left\{\mathrm{Rem}<ł|\mathrm{Rem}>0\right\}=\frac{P\left\{0<\mathrm{Rem}<ł\right\}}{P\left\{\mathrm{Rem}>0\right\}}=\frac{\Phi \left(\frac{ł-m_{\mathrm{Rem}}}{\sigma_{\mathrm{Rem}}}\right)-\Phi \left(-\frac{m_{\mathrm{Rem}}}{\sigma_{\mathrm{Rem}}}\right)}{1-\Phi \left(-\frac{m_{\mathrm{Rem}}}{\sigma_{\mathrm{Rem}}}\right)},$$

where Φ(·) stands for the CDF (the cumulative distribution function) of the standard normal distribution and ł>0.

Moreover, the joint condition meets the standard if their number does not deviate from the mean value by more than h times the standard deviation, where h∈[1,3]. The norms for the joint condition are therefore determined by the following inequalities:

- the joint for recycling Rec: $\left[m_{\mathrm{Rec}}-{h\sigma }_{\mathrm{Rec}},m_{\mathrm{Rec}}+{h\sigma }_{\mathrm{Rec}}\right],$
- the joint for reusing Reu: $\left[m_{\mathrm{Reu}}-{h\sigma }_{\mathrm{Reu}},m_{\mathrm{Reu}}+{h\sigma }_{\mathrm{Reu}}\right],$
- the joint for remanufacturing Rem: $\left[m_{\mathrm{Rem}}-{h\sigma }_{\mathrm{Rem}},m_{\mathrm{Rem}}+{h\sigma }_{\mathrm{Rem}}\right]\;\mathrm{or}$
- the joint for Waste: $\left[m_{\mathrm{Waste}}-{h\sigma }_{\mathrm{Waste}},m_{\mathrm{Waste}}+{h\sigma }_{\mathrm{Waste}}\right]$.

The quality of each joint $n_{j}^{i}$ of disassembled product i on r historical periods is investigated, namely in [(q−1)D, qD), where q=1, …, r. In each period q, in each cycle (every $\frac{D^{i}}{Z^{i}}$ time units) information on the joint $n_{j}^{i}$ quality for Rec, Reu, Rem or Waste is collected. For example, for the period [0,D) we collect information at times

$$0,\frac{D}{Z^{i}},\;\frac{2D}{Z^{i}},\;\ldots ,\;\frac{\left(Z^{i}-1\right)D}{Z^{i}},$$

where $Z^{i}$ is a number of cycles equal to the batch size of product i.

In a given period [(q−1)D, qD), the first moment of the evaluation is searched for, when the joint is outside the norm (normally qualified as Reu, Rem or Rec but not Waste). Introduce the below equations:

$$e_{s}^{\left(\mathrm{Rec}\right)}=\frac{\mathrm{number}\;\mathrm{of}\;\mathrm{cycles}\;\mathrm{in}\;\mathrm{which}\;\mathrm{the}\;\mathrm{joint}\;\mathrm{for}\;\mathrm{Rec}\;\mathrm{is}\;\mathrm{out}\;\mathrm{of}\;\mathrm{the}\;\mathrm{norm}\;\mathrm{at}\;\mathrm{the}\;\mathrm{sth}\;\mathrm{measurement}\;\mathrm{for}\;\mathrm{the}\;\mathrm{first}\;\mathrm{time}}{r},$$

$$e_{s}^{\left(\mathrm{Reu}\right)}=\frac{\mathrm{number}\;\mathrm{of}\;\mathrm{cycles}\;\mathrm{in}\;\mathrm{which}\;\mathrm{the}\;\mathrm{joint}\;\mathrm{for}\;\mathrm{Reu}\;\mathrm{is}\;\mathrm{out}\;\mathrm{of}\;\mathrm{the}\;\mathrm{norm}\;\mathrm{at}\;\mathrm{the}\;\mathrm{sth}\;\mathrm{measurement}\;\mathrm{for}\;\mathrm{the}\;\mathrm{first}\;\mathrm{time}}{r},$$

and

$$e_{s}^{\left(\mathrm{Rem}\right)}=\frac{\mathrm{number}\;\mathrm{of}\;\mathrm{cycles}\;\mathrm{in}\;\mathrm{which}\;\mathrm{the}\;\mathrm{joint}\;\mathrm{for}\;\mathrm{Rem}\;\mathrm{is}\;\mathrm{out}\;\mathrm{of}\;\mathrm{the}\;\mathrm{norm}\;\mathrm{at}\;\mathrm{the}\;\mathrm{sth}\;\mathrm{measurement}\;\mathrm{for}\;\mathrm{the}\;\mathrm{first}\;\mathrm{time}}{r},$$

where $s=0,\;\ldots ,{\;Z}^{i}-1$ (the measurement at (q−1)D is identified as the 0th one).

So, $e_{s}^{\left(\mathrm{Rec}\right)},{\;e}_{s}^{\left(\mathrm{Reu}\right)}$, $e_{s}^{\left(\mathrm{Rem}\right)}$ or $e_{s}^{\left(\mathrm{Waste}\right)}\;$are empirical probabilities where the first “outlier” (in measurement of parameter for Rec,Reu and Rem) is detected at the sth measurement in a single available period [(q−1)D, qD), respectively.

Finally, we can estimate the probability of a joint condition in the next available period [rD, (r+1)D) due to the “outlier” in the possible parameter value qualifying disassembly joint variants for Rec, Reu, Rem or Waste, respectively, after time ł∈[0, D) as

$$\sum_{s=\lfloor ł\rfloor +1}^{Z^{i}-1}e_{s}^{\left(\mathrm{Rec}\right)},\;\sum_{s=\lfloor ł\rfloor +1}^{Z^{i}-1}e_{s}^{\left(\mathrm{Reu}\right)},\;\sum_{s=\lfloor ł\rfloor +1}^{Z^{i}-1}e_{s}^{\left(\mathrm{Rem}\right)},\;\sum_{s=\lfloor ł\rfloor +1}^{Z^{i}-1}e_{s}^{\left(\mathrm{Waste}\right)}\;,$$

respectively.

### 3.3. Estimation of the Disassembly System Efficiency

The duration of the disassembly of joint j of product i is estimated using mean value $E_{j}^{i}\left(X_{q,k}\right)$ (10) typical for one of the four possible variants: Rec, Reu Rem and Waste. If the probability of the disassembly variant of Rec, Reu or Rem is within the norm, then the probability of executing a single joint of product after time ł∈[0, Ł) in the next available period can be expressed as

(19) $${\mathrm{Srec}}_{j}^{i}\left(ł\right)≝P\left\{\;\mathrm{joint}\;j\;\mathrm{of}\;\mathrm{product}\;i\;\mathrm{for}\;\mathrm{recycling}\;\mathrm{after}\;\mathrm{time}\;ł\right\}=1-\overset{k-1}{\underset{s=\lfloor ł\rfloor +1}{\sum }}e_{s}^{\left(\mathrm{Rec}\right)},$$

(20) $${\mathrm{Sreu}}_{j}^{i}\left(ł\right)≝P\left\{\;\mathrm{joint}\;j\;\mathrm{of}\;\mathrm{product}\;i\;\mathrm{for}\;\mathrm{reusing}\;\mathrm{after}\;\mathrm{time}\;ł\right\}=1-\overset{k-1}{\underset{s=\lfloor ł\rfloor +1}{\sum }}e_{s}^{\left(\mathrm{Reu}\right)}$$

(21) $${\mathrm{Srem}}_{j}^{i}\left(ł\right)≝\;P\left\{\;\mathrm{joint}\;j\;\mathrm{of}\;\mathrm{product}\;i\;\mathrm{ffot}\;\mathrm{facturingor}\;\mathrm{remanufacturing}\;\mathrm{after}\;\mathrm{time}\;ł\right\}\;\;\;\;\;\;\;\;\;\;\;\;\;=1-\sum_{s=\lfloor ł\rfloor +1}^{k-1}e_{s}^{\left(\mathrm{Rem}\right)},$$

where $j=1,\;\ldots ,{\;a}^{i}$ and 0≤ł<Ł.

The probability of qualifying joint j of product i as waste is given by analogy.

Disassembly operations with predicted duration are assigned to stations using the ranked positional weight (RPW) method or the largest candidate rule (LCR) method. In the LCR method, disassembly operations are allocated to stations based on the standard time length, the sequence of items and the required templates and tools [35]. In the RPW method, elements are assigned to a station on the basis of the RPW weight and their position in the precedence diagram [36]. The line efficiency (LE), smoothness index (SI) and line time (T) [37] are computed in order to assess the quality of the achieved solutions. A value of 100% is assumed to be ideal for the line efficiency:

(22) $${\mathrm{LE}}^{i}=\frac{\sum_{s=1}^{b^{i}}{\mathrm{ST}}_{s}^{i}}{C^{i}\times b^{i}}\times 100\%$$

where $b^{i}$—number of disassembly stations assigned for product i; $C^{i}$—cycle time computed for product i; ${\mathrm{ST}}^{i}$—the utilization time of station s assigned to product i.

The smaller the value of the line smoothness index, the better the line balancing. A value of 0 is ideal for the smoothness index:

(23) $${\mathrm{SI}}^{i}=\sqrt{\overset{b}{\underset{s=1}{\sum }}{\left({\mathrm{ST}}_{\max}^{i}-{\mathrm{ST}}_{s}^{i}\right)}^{2}}$$

where ${\mathrm{ST}}_{\max}^{i}$—the maximum time of using stations by product i; ${\mathrm{ST}}_{s}^{i}$—the utilization time of station s by product i.

The line time factor depends on the number of stations; lower value means better line balance:

(24) $${\mathrm{LT}}^{i}=\left(b-1\right)\times C^{i}+{\mathrm{ST}}_{b}^{i}$$

where ${\mathrm{ST}}_{s}^{i}$—the time of using the last station, b by product i.

The OP operating profit of the disassembly line is maximized by the recovery of recyclable materials or semi-finished products for remanufacture (regeneration) or reuse:

(25) $${\mathrm{OP}}^{i}=\overset{w^{i}}{\underset{j=1}{\sum }}\left\{{\mathrm{dp}}_{j}^{i}-\left({\mathrm{dc}}_{j}^{i}\cdot t_{j}^{i}\right)\right\}.$$

where $w^{i}=a^{i}$ if joint j of product i is intended for recycling and $w^{i}\neq a^{i}$ if joint j of product i is dedicated for reuse or remanufacture.

Underestimated or overstated disassembly operation times affect timeliness of disassembly; therefore, the last actual cycle time and the predicted cycle time of the last station are compared to calculate ex post error costs (26):

(26) $${\mathrm{CT}}^{i}=\left|C_{B_{e},b}^{i}-\hat{C_{B_{q+b},b}^{i}}\right|\cdot {\mathrm{rct}}_{}^{i}.$$

where $C_{B_{e},b^{i}}^{i}$—the last actual cycle time at the last station b assigned to product i, $\hat{C_{B_{q+b},b^{i}}^{i}}—$the last predicted cycle time at the last station b assigned to product i.

After subtracting formula (26) from formula (25), we get the total profit TP achieved after disassembling the batch of product i.

## 4. Numerical Example and Discussion

**Figure 4:**
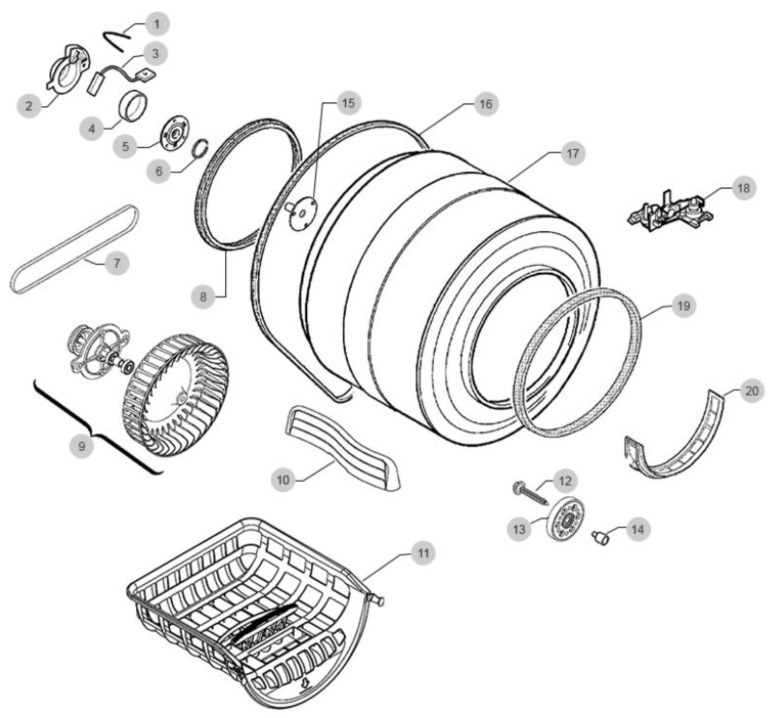
The diagram of North.pl dryer drum where: 1—Spring of the belt tensioner roller, 2—Bearing housing, 3—Motor carbon brush, 4—Engine sleeve, 5—Bearing, 6—Filter gasket, 7—Drive belt, 8—Back cover gasket, 9—Fan impeller, 10—Drum carrier, 11—Drying basket, 12—Bolt, 13—Belt tensioning roller, 14—Belt tensioner roller bushing, 15—Bearing axle, 16—Drive belt, 17—Complete drum, 18—Moisture sensor, 19—Gasket, 20—Engine mount.

Let us consider the disassembly line of the dryer drum; the structure of the dryer drum consists of 20 elements presented in Figure 4.

The matrix of constraints ${\mathrm{MP}}^{1}=p_{j,k}^{1}$ (Table 1) represents the relationships between the successive joints $n_{j}^{i}$ and $n_{k}^{i}$:

The transport time of the conveyor between the individual disassembly station TT^s^ does not disturb the cycle time. Batch size of the product Z^1^ is 7. The objective is to achieve the minimum number of disassembly stations s with the maximum line efficiency (22) and the maximum total profit TP achieved after the disassembly of the given batch size of the product. The matrix of disassembly operation times ${\mathrm{MT}}^{1}=t_{j}^{1}$ represents the predicted times obtained after the analysis of historical information on the times and quality of disassembly joints (Table 2). The cycle time C^1^ is 20 min.

Let us analyze how to achieve the predicted time for the complete drum, joint n_j_ = 17, based on historical quality information and disassembly times. The joint quality changes over time due to different mechanical and topological properties caused by wear, rust or deformation. It is necessary to predict the quality of the joint either for recycling (Rec), reusing (Reu), remanufacturing (Rem) or Waste in order to analyze relevant historical times for disassembly operations.

In a situation where it is difficult to identify the parameter classifying the joint either for recycling (Rec), reusing (Reu), remanufacturing (Rem) or as Waste, only the historical classification information is considered for analysis: the joint qualified for Rec, Reu, Rem or as Waste. The quality of the disassembled joint was monitored in each historical period q=1, …, 30. In each of available periods, for each cycle $k_{q}=$ {80,70,100,…,70} one piece of information was collected: the joint for Rec, Reu, Rem or for Waste.

Numerical analysis was performed with the application of the MS Excel. Empirical probabilities $q_{i}^{\left(F\right)}$ that the first “outlier” (normally the joint is qualified for Rec) occurs at the kth measurement in an available historical period [(q−1)D, qD) are

$$\begin{array}{cc} q_{1}^{\left(\mathrm{Rec}\right)}=0,q_{2}^{\left(\mathrm{Rec}\right)} & =0,q_{3}^{\left(\mathrm{Rec}\right)}=0.0333,q_{4}^{\left(\mathrm{Rec}\right)}=0.1,q_{5}^{\left(\mathrm{Rec}\right)}=0,q_{6}^{\left(\mathrm{Rec}\right)}=0.0667,q_{7}^{\left(\mathrm{Rec}\right)}=0,q_{8}^{\left(\mathrm{Rec}\right)}=0,q_{9}^{\left(\mathrm{Rec}\right)}\\ & =0,q_{10}^{\left(\mathrm{Rec}\right)}=0,q_{11}^{\left(\mathrm{Rec}\right)}=0,q_{12}^{\left(\mathrm{Rec}\right)}=0.1333,q_{13}^{\left(\mathrm{Rec}\right)}=0,q_{14}^{\left(\mathrm{Rec}\right)}=0,q_{15}^{\left(\mathrm{Rec}\right)}=0,q_{16}^{\left(\mathrm{Rec}\right)}=0,q_{17}^{\left(\mathrm{Rec}\right)}\\ & =0,q_{18}^{\left(\mathrm{Rec}\right)}=0,q_{19}^{\left(\mathrm{Rec}\right)}=0,q_{20}^{\left(\mathrm{Rec}\right)}=0.0333\ldots \end{array}$$

Consequently, we can estimate the probability of qualifying the joint for recycling in the next available period [rT, (r+1)T) due to the “outlier” in historical qualification data for Rec, after time ł∈[0, 25) as

$$\overset{70-1}{\underset{s=25+1}{\sum }}e_{s}^{\left(\mathrm{Rec}\right)}=8\%,\;$$

The probability of executing the joint of product for recycling after time ł∈[0, 25) in the next available period is expressed as

$${\mathrm{Srec}}_{j}^{i}\left(ł\right)≝P\left\{\mathrm{executing}\;\mathrm{joint}\;17\;\mathrm{of}\;\mathrm{the}\;\mathrm{product}\;\mathrm{for}\;\mathrm{recycling}\;\mathrm{after}\;\mathrm{time}\;25\right\}=\left(1-\sum_{s=25+1}^{70-1}e_{s}^{\left(\mathrm{Rec}\right)}\right)=92\%,$$

The historical data concerning the disassembly duration of the joint classified for Rec was collected (Table 3). The duration of the disassembly joint of the dryer drum was described by truncated normal distribution with parameters changing with time. Each historical available period equaled 480 min.

**Figure 5:**
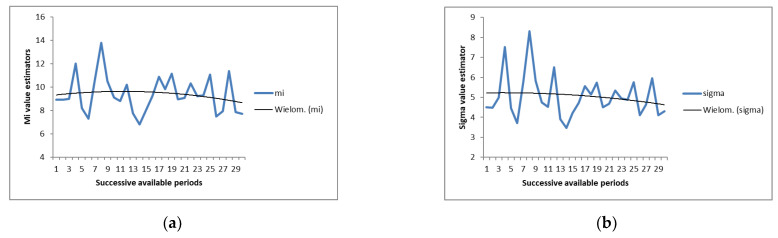
Values of estimators together with the fitted functions for: (**a**) m_1_,…, m_30_; (**b**) σ_1_,…, σ_30_.

Parameters m_1_,…, m_30_ and σ_1_,…, σ_30_ of normal distribution truncated to the positive half-axis described the disassembly time of the joint. The parameters of the normal distribution were estimated using Equations (13)–(15). Values of estimators m_1_,…, m_30_ and σ_1_,…, σ_30_ together with the fitted functions are plotted in Figure 5. After finding successive estimators m_1_,…, m_30_ and σ_1_,…, σ_30_, values of ${\hat{m}}_{31}$ and ${\hat{\sigma }}_{31}$ were estimated for the future period [30D, 31D] using the least squares method. The Equations used for the extrapolation of future estimators are presented in (Table 4).

The expected value (EX) of normal distributions truncated to the positive half-axis described by parameters achieved using the maximum likelihood method was 10.04.

**Figure 6:**
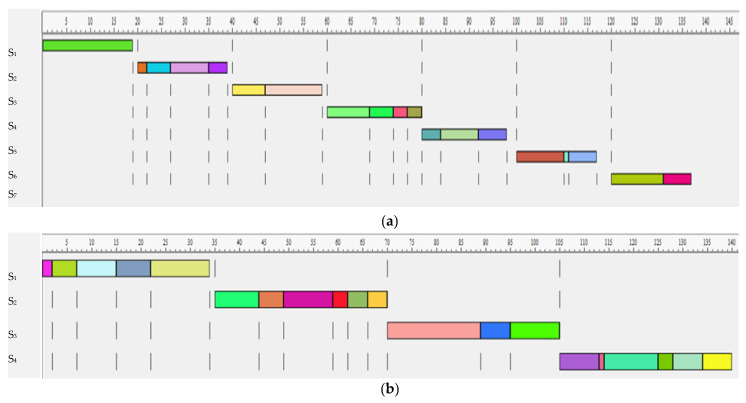
Disassembly tasks assigned to stations according to the decreasing demand and: (**a**) cycle time equals 20 min; (**b**) cycle time equals 35 min.

Using the largest candidate rule method, the operations were allocated according to the decreasing demand (number of predecessors), taking into account the matrix of the relationships between nodes (Table 1). This resulted in the following assignments of tasks: A1 = {16}, I1 = 20 − 19 = 1; A2 = {1,2,3,20}, I2 = 20 − 19 = 1; A3 = {4,5}, I3 = 20 − 19 = 1; A4 = {6,7,14,19}, I4 = 20 − 20 = 0; A5 = {12,13,18}, I5 = 20 − 18 = 2; A6 = {8,15,11}, I6 = 20 − 17 = 3; A7 = {9,10}, I7 = 20 − 17 = 3. The assignment of tasks is presented in the Gantt chart (Figure 6a):

Seven disassembly stations were required for the cycle time of 20 min. Assuming that the cycle time equals 35 min, four stations were required (Figure 6b). This resulted in the following assignments of tasks: A1 = {1,2,3,4,5}, I_1_ = 35 − 34 = 0; A2 = {6,7,8,14,12,20}, I_2_ = 35 − 35 = 0; A3 = {16,18,17}, I_3_ = 35 − 35 = 0; A4 = {13,15,10,19,11,9}, I_4_ = 35 − 35 = 0.

After applying the RPW (ranked positional weight) method, disassembly operations were assigned to stations on the basis of weights. The weight was calculated by summing up the time of a given operation and operations directly related to it, i.e., the predecessors. The line efficiency (LE), smoothness index (SI) and line time (T) [37] were computed in order to assess the quality of the achieved solutions using the methods LCR and RPW. The results are presented in Table 5.

In both methods, better line efficiency was achieved when the cycle time equaled 35. When the cycle time equaled 35, both methods achieved the same quality solutions, taking into account the line efficiency index (99.29%), smoothness index (1) and line time (140). The LCR method achieved better solutions, considering the line smoothness index and the cycle time equaled 20. The line time factor depended on the number of stations; thus, better line balance was achieved for the solution reached using the LCR method.

Assume that the conveyor transport was performed manually and that travel time was performed simultaneously within the cycle time and the third disassembly station was disturbed during the third batch of the product. Although the cycle time was one minute longer, the delay was compensated for by the idle time at station five for the solution achieved using the LCR and the cycle time equaled 20. The disassembly time of the 20 products is two minutes longer for the cycle time equaling 35.

The presented estimation method of disassembly operation times influences the reliability and efficiency of elaborated balances of tasks in lines. However, the effectiveness of the planned disassembly lines also depends on the method used to allocate operations to stations. Therefore, in the future, different methods of allocating operations to stations, based on artificial intelligence, will be developed and applied to investigate the best allocation method.

## 5. Conclusions

Serial disassembly sequence planning problems consist mostly of theoretical research and are not practically applied [31]. In the complete serial disassembly sequence planning, one component is disassembled at a time until the product is completely disassembled. In the selective serial disassembly sequence planning, when the target node (component) is reached, the selective disassembly task is completed and the refurbished component is repaired, reused or remanufactured. The article presents a disassembly planning method with three options: recycling, reuse or regeneration. The presented estimation method of disassembly operation times influences the reliability and efficiency of the elaborated balances of tasks in lines.

The presented method is planned to be developed with sequence-dependent setup times of disassembly batch production for transport operations, machine setup changes and tool changes. The sequence-dependent setup times influence disassembly systems, including the number of changes in the disassembly tool and direction of the operation, as is mentioned in [38]. Additionally, different methods of allocating operations to stations, based on artificial intelligence, will be developed and applied to investigate the best allocation method.

## Figures and Tables

**Table 1:** The matrix of the relationships between nodes of the dryer drum.

p^1^_jk_	1	2	3	4	5	6	7	8	9	10	11	12	13	14	15	16	17	18	19	20
1	0	−x	−x	−x	−x	−x	1	−x	0	0	0	1	1	1	−x	0	0	0	0	0
2	0	0	−x	−x	−x	−x	1	−x	0	0	0	1	1	1	−x	0	0	0	0	0
3	0	0	0	−x	−x	−x	1	−x	0	0	0	1	1	1	−x	0	0	0	0	0
4	0	0	0	0	−x	−x	1	−x	0	0	0	1	1	1	−x	0	0	0	0	0
5	0	0	0	0	0	−x	1	−x	0	0	0	1	1	1	−x	0	0	0	0	0
6	0	0	0	0	0	0	1	−x	0	0	0	1	1	1	−x	0	0	0	0	0
7	0	0	0	0	0	0	0	1	1	0	0	1	1	1	1	0	0	0	0	0
8	0	0	0	0	0	0	0	0	0	0	0	0	0	0	−x	0	0	0	0	0
9	0	0	0	0	0	0	0	0	0	0	0	0	0	0	0	0	0	0	0	0
10	0	0	0	0	0	0	0	0	0	0	0	0	0	0	0	0	0	0	0	0
11	0	0	0	0	0	0	0	0	0	0	0	0	0	0	0	0	0	0	0	0
12	0	0	0	0	0	0	0	0	0	0	0	0	−x	0	0	0	0	0	0	0
13	0	0	0	0	0	0	0	0	0	0	0	0	0	0	0	0	0	0	0	0
14	0	0	0	0	0	0	0	0	0	0	0	x	x	0	0	0	0	0	0	0
15	0	0	0	0	0	0	0	0	0	0	0	0	0	0	0	0	0	0	0	0
16	0	0	0	0	0	0	0	0	0	1	1	0	0	0	0	0	0	1	0	0
17	0	0	0	0	0	0	0	0	0	0	0	0	0	0	0	0	0	0	0	0
18	0	0	0	0	0	0	0	0	0	0	0	0	0	0	0	0	0	0	0	0
19	0	0	0	0	0	0	0	0	0	0	0	0	0	0	0	0	0	0	0	0
20	0	0	0	0	0	0	0	0	0	0	0	0	0	0	0	0	0	0	x	0

**Table 2:** The matrix of disassembly operation times of the dryer drum joints.

t^1^_j_	1	2	3	4	5	6	7	8	9	10	11	12	13	14	15	16	17	18	19	20
1	2	5	8	7	12	9	5	10	6	11	6	4	8	3	1	19	10	6	3	4

**Table 3:** Disassembly times $t_{j}^{1}$ of the joint qualified for Rec collected during available periods (a shift), q = 1,2,…,30.

The Number of Cycle Times (and Disassembly Times of the Joint for Rec) in Available Periods q				
9(9,9,10,11,10,	7(10,10,10,	9(11,9,10,12,	6(10,12,14,	10(9,13,13,9,
11,11,13,12)	12,12,10,11)	12,10,11,13,12)	16,18,20)	7,8,12,9,8,10)
11(10,9,9,9,9	8(10,14,15,	6(10,10,15,15,	8(20,10,12,	9(9,13,10,9,
,9,8,8,9,9,8)	14,18,10,10,8)	28,17)	13,15,9,10,9)	13,12,13,9,10)
9(8,9,10,11,12,	7(10,12,12,13,	10(11,9,9,9,8,	11(10,8,8,8,	10(9,9,10,10,
12,12,10,11)	14,15,16)	10,9,10,9,9)	8,8,9,9,8,7,7)	9,15,8,9,8,8)
9(8,9,10,11,12,	7(10,12,12,13,	8(9,9,10,11,12,	7(10,11,12,	9(12,11,10,10,
12,12,12,13)	14,14,16)	12,14,16)	14,15,15,16)	11,10,10,11,12)
9(8,8,10,11,11,	8(8,10,12,12,13,	9(7,8,8,9,11,	9(7,8,10,11,	7(10,11,12,
11,12,12,14)	14,14,15)	12,13,14,15)	11,12,13,13,14)	13,14,14,18)
11(5,6,6,7,8,8,	10(5,6,6,6,8,9,	7(9,10,13,14,	10(7,7,8,8,9,	11(5,5,6,8,8,9,
10,11,11,12,12)	11,12,14,14)	15,16,17)	9,10,11,12,12)	9,10,12,13,13)

**Table 4:** Calculation of distribution parameters ${\hat{m}}_{31}$, ${\hat{\sigma }}_{31}$ describing the disassembly times of the joint predicted for Rec.

Maximum Likelihood Approach		
Parameters	Function Decribing the Parameters	Values
${\hat{m}}_{31}$	y = −0.0028x^2^ + 0.064x + 9.2538	8.547
${\hat{\sigma }}_{31}$	y = −0.0009x^2^ + 0.0062x + 5.2185	4.5458

**Table 5:** Comparative analysis of the methods: largest candidate rule and ranked positional weight to balance the disassembly line dedicated to the complete drum.

Method	Cycle Time	No Stations	Line Efficiency (LE)	Smoothness Index (SI)	Line Time (T)
LCR	20	7	92.14%	5.10	137
	35	4	99.29%	1	140
RPW	20	8	86.88%	14.46	146
	35	4	99.29%	1	140

## Data Availability

Not applicable.
